# A united risk model of 11 immune‑related gene pairs and clinical stage for prediction of overall survival in clear cell renal cell carcinoma patients

**DOI:** 10.1080/21655979.2021.1955558

**Published:** 2021-07-24

**Authors:** Zijia Tao, Enchong Zhang, Lei Li, Jianyi Zheng, Yiqiao Zhao, Xiaonan Chen

**Affiliations:** Department of Urology, Shengjing Hospital of China Medical University, Shenyang, Liaoning, People’s Republic of China

**Keywords:** Clear cell renal cell carcinoma, immune-related gene pairs, prognostic signature, the cancer genome atlas, arrayexpress, IRGPs

## Abstract

Clear cell renal cell carcinoma (ccRCC) is the most common subtype of renal cancer. Currently, we lack effective risk models for the prognosis of ccRCC patients. Given the significant role of cancer immunity in ccRCC, we aimed to establish a novel united risk model including clinical stage and immune-related gene pairs (IRGPs) to assess the prognosis. The gene expression profile and clinical data of ccRCC patients from The Cancer Genome Atlas and Arrayexpress were divided into training cohort (n = 381), validation cohort 1 (n = 156), and validation cohort 2 (n = 101). Through univariate Cox regression analysis and Least Absolute Shrinkage and Selection Operator analysis, 11 IRGPs were obtained. After further analysis, it was found that clinical stage could be an independent prognostic factor; hence, we used it to construct a united prognostic model with 11 IRGPs. Based on this model, patients were divided into high-risk and low-risk groups. In Kaplan–Meier analysis, a significant difference was observed in overall survival (OS) among all three cohorts (p < 0.001). The calibration curve revealed that the signature model is in high accordance with the observed values of each data cohort. The 1-year, 3-year, and 5-year receiver operating characteristic curves of each data cohort showed better performance than only IRGP signatures. The results of immune infiltration analysis revealed significantly (p < 0.05) higher abundance of macrophages M0, T follicular helper cells, and other tumor infiltrating cells. In summary, we successfully established a united prognostic risk model, which can effectively assess the OS of ccRCC patients.

## Graphical Abstract

**Figure:**
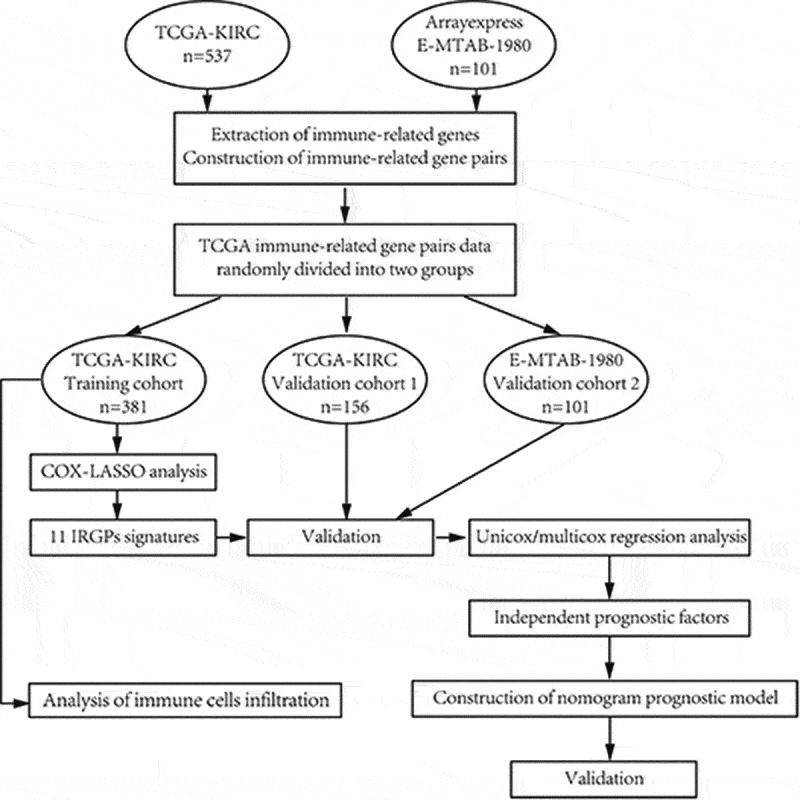

## Introduction

Renal cell carcinoma (RCC) is the 14^th^ most common cancer in the world, with a higher incidence in males than females [1]. In the past 20 years, the incidence of RCC has increased annually [2]. According to the 2016 classification of World Health Organization (WHO), there are three main subcategories of RCC: (1) clear cell renal cell carcinoma (ccRCC), which is the most common type; (2) papillary renal cell carcinoma (pRCC); and (3) chromophobe renal cell carcinoma (chRCC) [3,4]. In this study, we focused on ccRCC. Currently, surgery is the most recommended treatment for localized ccRCC [5]. However, approximately one-third of patients relapse [6]. In addition, because of the location of the kidney in the body, many patients remain asymptomatic until the kidney mass develops to an advanced stage [3,4]. These factors have also led to only a small improvement in the prognosis of ccRCC in the past two decades [7–11]. Therefore, it is very important to explore the detailed mechanisms underlying ccRCC and improve the prognosis of patients.

The significant role of immune system in cancer has been proved previously [12]. The immune factors, immune cells, and immune microenvironment are essential factors for tumorigenesis [13]. All cancers can be considered immunogenic to some extent, and the immune system of the host can produce T cell responses, which can identify and eliminate cancer cells [14]. In addition, tumor-associated immunity is present in all stages of tumorigenesis [15]. In view of the complexity of the tumor immune microenvironment, studying the immune-related genes (IRGs) of ccRCC patients is imperative. In the past two years, some researchers have studied the prognostic value and role of IRGs in RCC. They have constructed a prognostic model based on the expression of IRGs by analyzing transcriptome data on ccRCC in TCGA. However, this platform lacks validation data from other platforms and it has platform bias [16]. There is also a study based on the bioinformatic analysis of pRCC in which a prognostic model was constructed based on immune-related gene pairs (IRGPs), but this model does not include clinical factors [17]. Therefore, we need to build a comprehensive and accurate immune-related gene prognostic evaluation system for ccRCC.

At present, studies have proposed signatures based on gene expression, which can be applied to assess the prognosis of patients with kidney cancer [16,18]. Due to the technology biases among different sequencing platforms or potential biological heterogeneity between datasets, previous studies that used expression levels of genes need proper normalization, which brings some difficulties to data processing [19]. In addition, because of the overfitting of small data cohorts and lack of enough verification, these signatures and models have not been applied to routine clinical practice. In recent years, some researchers have proposed a new method based on gene-expression relative ranking, which can avoid the drawbacks of scaling of gene expression data and normalization, and reliable results and conclusions have been achieved with this method in many studies [20–22].

In order to overcome the abovementioned problems and obtain a reliable prognostic evaluation system for ccRCC, according to the information from IRGs from the ImmPort database, we divided two RNA-seq datasets into a training cohort, an internal validation cohort, and an external validation cohort, and established and verified a risk model of 11 IRGPs in patients with ccRCC. Then, we combined it with the stage of clinicopathological factors to construct a comprehensive prognostic signature model.

## Materials & methods

### Data acquisition

**Figure 1.**
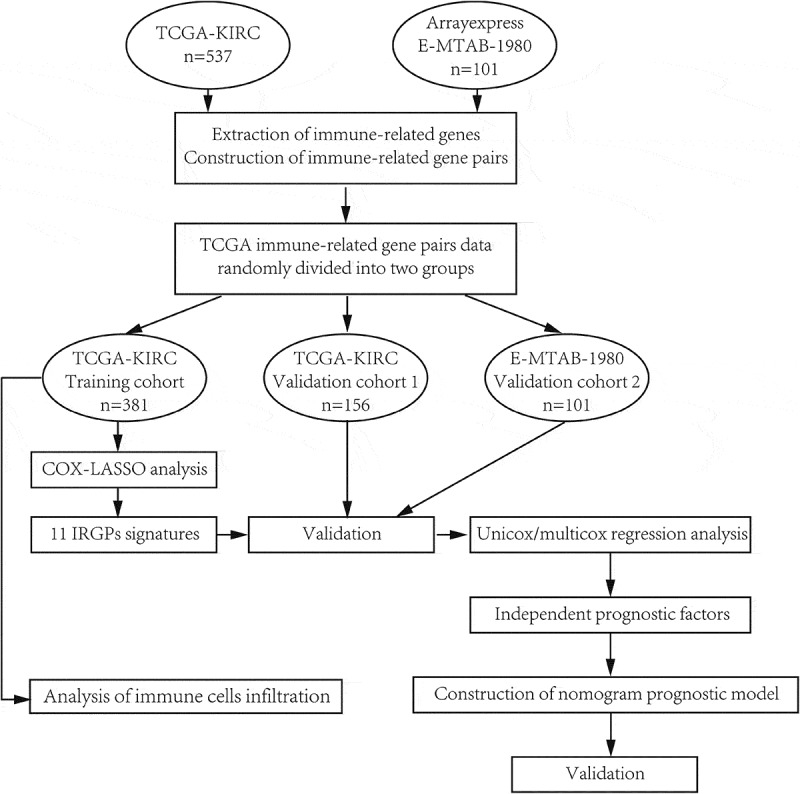
Overall workflow of this study

We obtained gene expression information (FPKM) and matched ccRCC patients’ clinical data from TCGA dataset (https://cancergenome.nih.gov/). In addition, gene expression and clinical information of ccRCC patients were also downloaded from the E-MTAB-1980 of ArrayExpress (https://www.ebi.ac.uk/arrayexpress/). They are publicly available and unrestricted re-use is permitted via an open license. Log2 processing was performed on gene expression data of E-MTAB-1980 to make it in the same order of magnitude as the expression data from TCGA. All patient records with incomplete information were deleted. The detailed overall workflow is presented in Figure 1.

### Construction of immune-related gene pairs (IRGPs)

An IRG list was achieved from the Immport Shared Gene Lists Data (https://www.immport.org). Then, the expression data of IRGs from TCGA and E-MTAB-1980 were extracted to a new matrix based on the list separately. The IRGs measured on different platforms with higher variability were selected for our research (screened based on the median absolute deviation >0.5). Then, based on the two IRGs matrices obtained in the previous step, we constructed respective IRGPs and analyzed their intersection. IRGs were matched to compare with each other to obtain IRGPs. If the first IRG’s expression level was higher than the second one, their IRGP value was 1; otherwise, the value was 0^17^. In TCGA and E-MTAB-1980, IRGPs with an IRGP score of 0 or 1 and a ratio of less than 80% were retained as candidate IRGPs for prognostic prediction.

### Preparation of training cohort and validation cohorts

The data of TCGA IRGPs were randomly divided into two groups. The ratio of the number of patients between the two groups was 7:3. The group with a large number of patients was defined as training cohort, and the other one was defined as validation cohort 1. Using Microsoft Office Excel software (version Professional Plus 2016), each patient was assigned a random number which was greater than 0 but less than 1. If the random number was greater than 0.7, the related patient was assigned to validation cohort 1, and if the random number was less than or equal to 0.7, the related patients were assigned to training cohort. The E-MTAB-1980 IRGP matrix was used as validation cohort 2.

### Construction of IRGP prognostic signature

In the training cohort, each IRGP was subjected to univariate Cox regression analysis, and the ‘coxph’ function of R (3.6.1) package ‘survival’ was used to analyze data, with the filter condition p < 0.001. After single factor Cox regression analysis, we applied the Least Absolute Shrinkage and Selection Operator (LASSO) algorithm (iteration = 10,000) to construct a concise and informative model. The risk score of IRGPs was calculated as follows: Risk score = (Exp_genepair1_ × Coef_genepair1_) + (Ex p_genepair2_ × Coef_genepair2_) + … + (Exp_genepairn_ × Coef_genepairn_). Here, ‘Exp’ is the value of IRGP and ‘Coef’ is the coefficient achieved from the LASSO algorithm. Based on the scores calculated by the risk characteristic signature, patients in training cohort, validation cohort 1, and validation cohort 2 were assigned to high-risk and low-risk groups separately, and the median score was used as a cutoff value [23].

### Validation of the IRGP risk model

In the three cohorts, the test efficiency of IRGP risk model was validated through receiving operator characteristic (ROC) curve analysis and 1-year, 3-year, and 5-year area under curve (AUC) values, and AUC>0.7 was considered as an ideal result [24]. We applied Kaplan–Meier (KM) survival curve to analyze the OS of the high-risk and low-risk groups, with the criterion of significance set at p < 0.05. Then, univariate Cox regression analysis was performed on IRGPs in training cohort and existing clinicopathological characteristics, including five indicators: gender, age, stage, grade, and risk score, and then indicators with p-value less than or equal to 0.001 were selected for multivariate Cox regression analysis. The univariate and multivariate Cox regression analyses were also performed in two validation cohorts. In addition, we explored the difference in IRGP score between Stage1-2 and Stage3-4, as well as the difference in IRGP score between Grade1-2 and Grade3-4.

### Construction of nomogram prognostic model

We further used the significant indicators with p-value less than 0.05 in the multivariate Cox regression analysis to construct a nomogram model as a united risk signature composed of independent prognostic factors. Based on the scores calculated through the nomogram prognostic model, three cohorts were allocated to low-risk and high-risk groups separately, and the median score was used as a cutoff value [25].

### Verification of the united risk model

To validate the test efficiency of the model, the consistency index (C-index) was calculated, and C-index>0.7 was considered as an ideal result. In addition, we also drew calibration plots for three years. In the three cohorts, the test efficiency of IRGP risk model was validated through ROC curve analysis and 1-year, 3-year, and 5-year AUC values, AUC >0.7 was considered as an ideal result. Besides, KM analysis was performed to analyze the OS of low-risk and high-risk groups, with the criterion of significance set at p < 0.05^25^.

### Analysis of immune cell infiltration between two risk groups

We applied CIBERSORT to assess the status of immune cell infiltration in the training cohort to determine the difference in infiltration status in ccRCC between the two risk groups. CIBERSORT is a software for deconvolution of the immune cell subtypes matrix according to the rule of linear support vector regression [26]. The RNA-seq data of the training cohort was used to estimate immune cell infiltration. After deleting invalid data, we analyzed the relative abundance of 21 infiltrating immune cells, including B cells, NK cells, and T cells.

### Statistical analysis

We used R (3.6.1) for statistical analysis. ‘glmnet’ software package was used to perform LASSO algorithm. The ROC curve was generated through ‘survivalROC’ package. The survival curve was obtained using the ‘survminer’ package. The ‘rms’ package was used to obtain the C-index and to generate the calibration curve. The criterion of significance was p < 0.05 for all tests. * P < 0.05, ** P < 0.01, *** P < 0.001 indicate different levels of statistical significance.

## Results

We aimed to establish a novel united risk model including clinical stage and IRGPs to assess the prognosis of patients with ccRCC. The gene expression profile and clinical data of ccRCC patients from TCGA and ArrayExpress were divided into training cohort (n = 381), validation cohort 1 (n = 156), and validation cohort 2 (n = 101). Through univariate Cox regression analysis and LASSO analysis, 11 IRGPs were obtained. After further analysis, it was found that clinical stage could be an independent prognostic factor; hence, we used it to construct a united prognostic model with 11 IRGPs. Based on this model, patients were divided into high-risk and low-risk groups. In KM analysis, a significant difference was observed in OS between the two groups (p < 0.001). The calibration curve revealed that the signature model is in high accordance with the observed values of each data cohort. The 1-year, 3-year, and 5-year ROC curves of each data cohort showed better performance than only IRGP signatures. The results of immune infiltration analysis revealed significantly higher abundance of macrophages M0, T cells follicular helper, and other tumor infiltrating cells. In summary, we successfully established a united prognostic risk model, which can effectively assess the OS of ccRCC patients.

### Establishment of the IRGP prognostic signature

**Figure 2.**
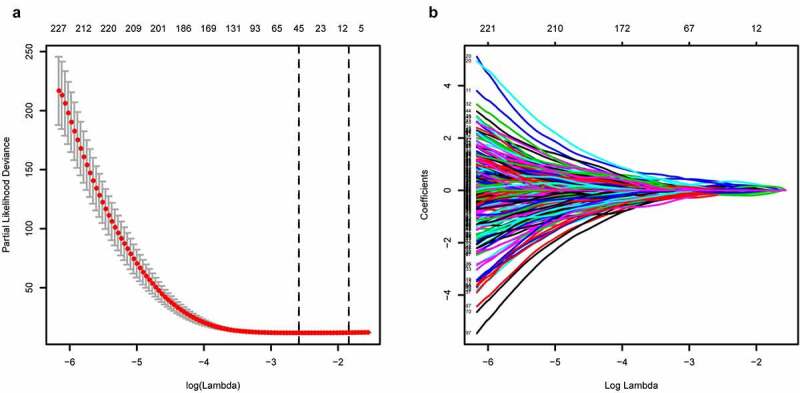
Predictor selection by the LASSO

We downloaded whole transcriptome expression data and clinical information of 537 and 101 patients from the official websites of TCGA and Arrayexpress, respectively. In all, 2498 IRGs were obtained from the Immport website, and 29,991 IRGPs were generated through gene pairwise calculation in TCGA and E-MTAB-1980. Then, we split TCGA data into training cohort (n = 381) and validation cohort 1 (n = 156) and used E-MTAB-1980 as validation cohort 2 (n = 101). All patient records with incomplete information were deleted (Table 1). We performed univariate Cox regression analysis on 29,991 IRGPs and found 5198 IRGPs with prognostic potential (p < 0.001). After that, LASSO analysis was conducted to simplify our risk model (Figure 2), and 11 IRGPs were selected for further research (Table 2).

**Table 1.** Available clinical and pathologic factors of the cohorts used in this study

	Training cohort (TCGA-KIRC)	Validation cohort1 (TCGA-KIRC)	Validation cohort2 (Arrayexpress, E-MTAB-1980)
Age			
<60	166 (45.6%)	75 (50.0%)	40 (40.4%)
≥60	198 (54.4%)	75 (50.0%)	59 (59.6%)
Gender			
Male	244 (67.0%)	92 (61.3%)	76 (76.8%)
Female	120 (33.0%)	58 (38.7%)	23 (23.2%)
Grade			
I	8 (2.2%)	5 (3.3%)	13 (13.1%)
II	158 (43.4%)	66 (44.0%)	59 (59.6%)
III	143 (39.3%)	61 (40.7%)	22 (22.2%)
IV	55 (15.1%)	18 (12.0%)	5 (5.1%)
Stage			
I	182 (50.0%)	75 (50.0%)	66 (66.7%)
II	37 (10.1%)	16 (10.7%)	8 (8.1%)
III	81 (22.3%)	41 (27.3%)	13 (13.1%)
IV	64 (17.6%)	18 (12.0%)	12 (12.1%)
Survival status			
Alive	245 (67.3%)	107 (71.3%)	76 (76.8%)
Dead	119 (32.7%)	43 (28.7%)	23 (23.2%)

**Table 2.** Information of the 11 immune-related gene pairs and their coefficient

Gene pair1	Immune process	Gene pair2	Immune process	Coefficient
PSMD11	Antigen_Processing_and_Presentation	NFKB1	Antimicrobials/BCRSignalingPathway/TCRsignalingPathway	0.0114928716374979
PSMD11	Antigen_Processing_and_Presentation	F2RL1	Antimicrobials	0.150699162881823
SLC10A2	Antigen_Processing_and_Presentation	AGER	Antimicrobials	−0.0350143079368729
CXCL2	Antimicrobials/Chemokines/Cytokines	GMFB	Cytokines	0.010646975575061
IL6	Antimicrobials/Chemokines/Cytokines	TGFB2	Cytokines/TGFb_Family_Member	0.0131725045908718
TLR7	Antimicrobials	IL20RB	Cytokine_Receptors/Interleukins_Receptor	−0.132192547462545
TYK2	Antimicrobials	KL	Cytokines	0.0225301659700258
IRF9	Antimicrobials	AR	Cytokine_Receptors	0.123358847050174
BIRC5	Antimicrobials	AR	Cytokine_Receptors	0.0167267736278587
PTK2	Antimicrobials	PLCG1	NaturalKiller_Cell_Cytotoxicity/TCRsignalingPathway	−0.0485464799522758
PLAUR	Chemokine_Receptors/Cytokine_Receptors	TEK	Cytokine_Receptors	0.154162736753599

(a) Parameter (Lambda) selection by LASSO model. (b) LASSO coefficient profile plot of variables against the log (Lambda) sequence.

### Validation of the IRGP signature

**Figure 3.**
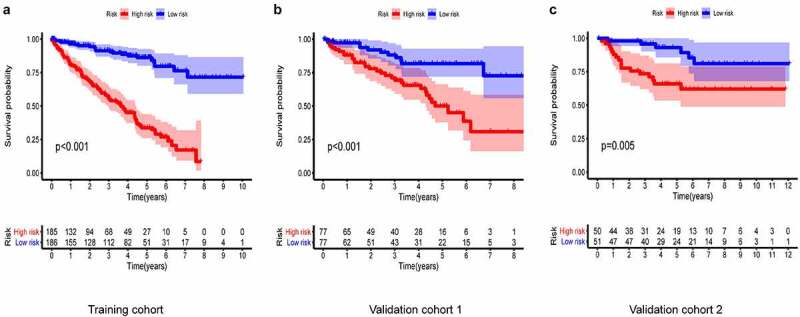
Survival plot of the three cohorts

**Figure 4.**
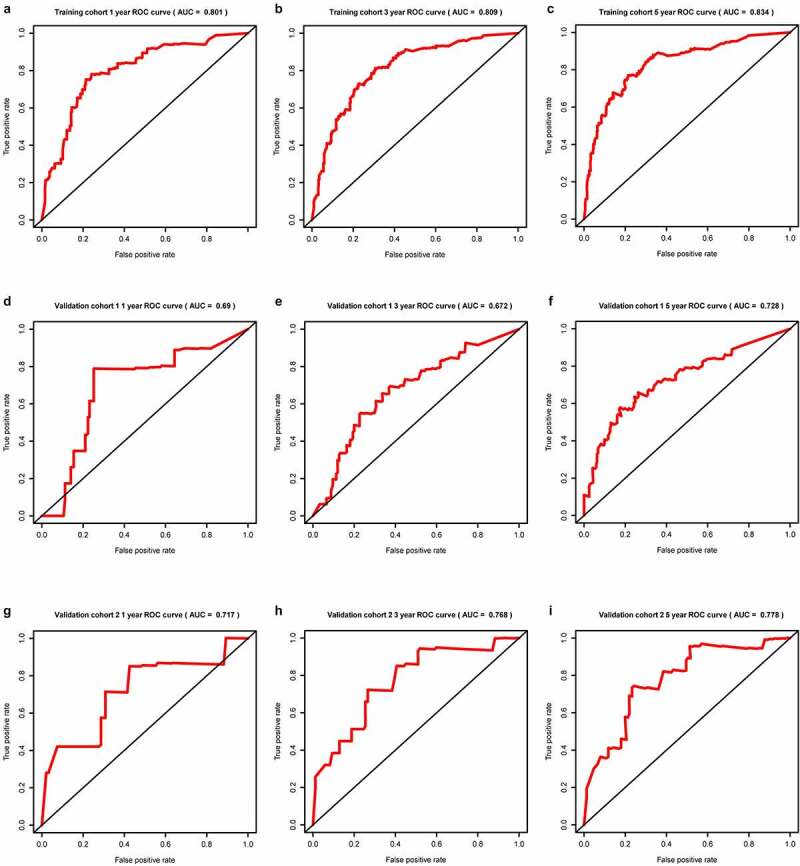
1, 3, and 5 year ROC curve of the three cohorts

**Figure 5.**
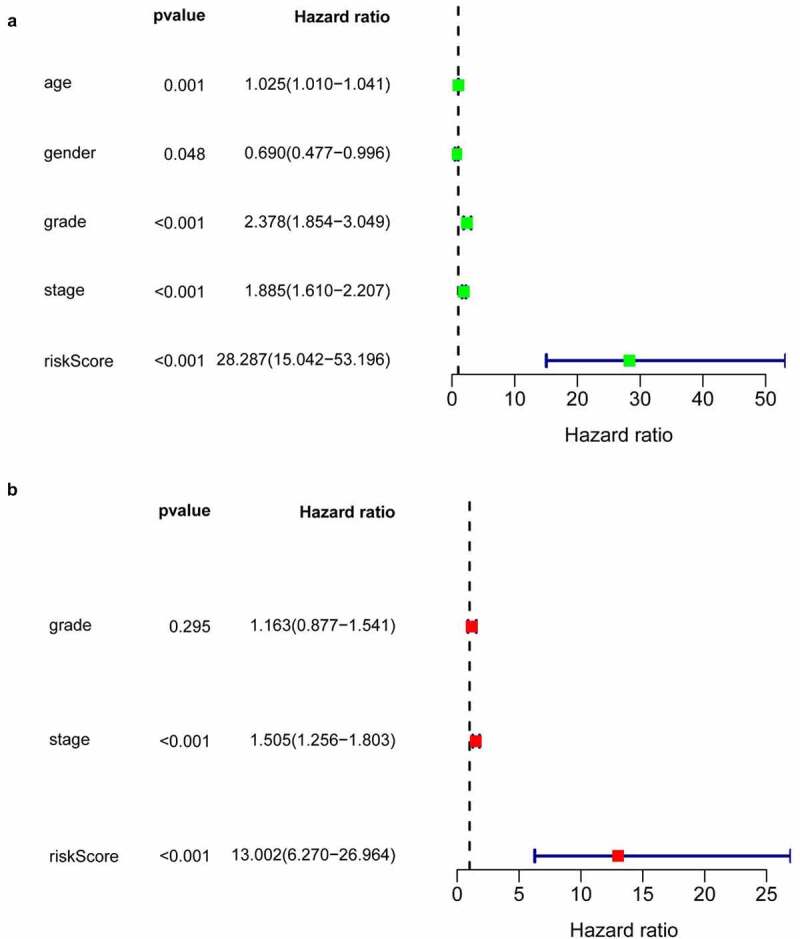
Univariate and multivariate Cox regression analysis forest plot of training cohort

**Figure 6.**
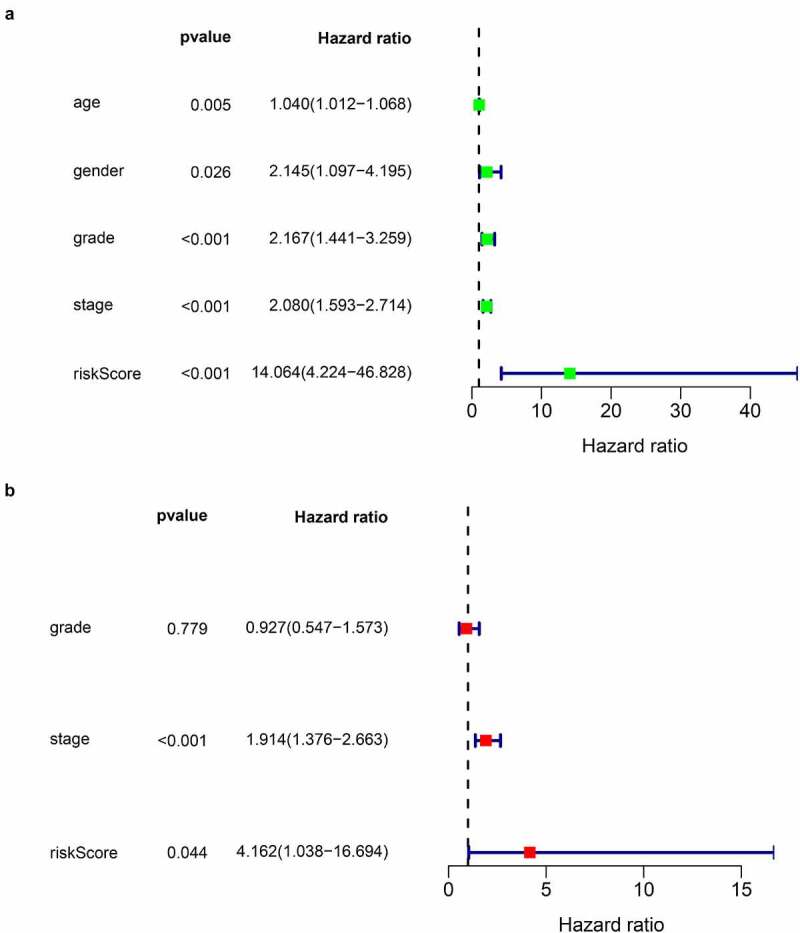
Univariate and multivariate Cox regression analysis forest plot of validation cohort 1

**Figure 7.**
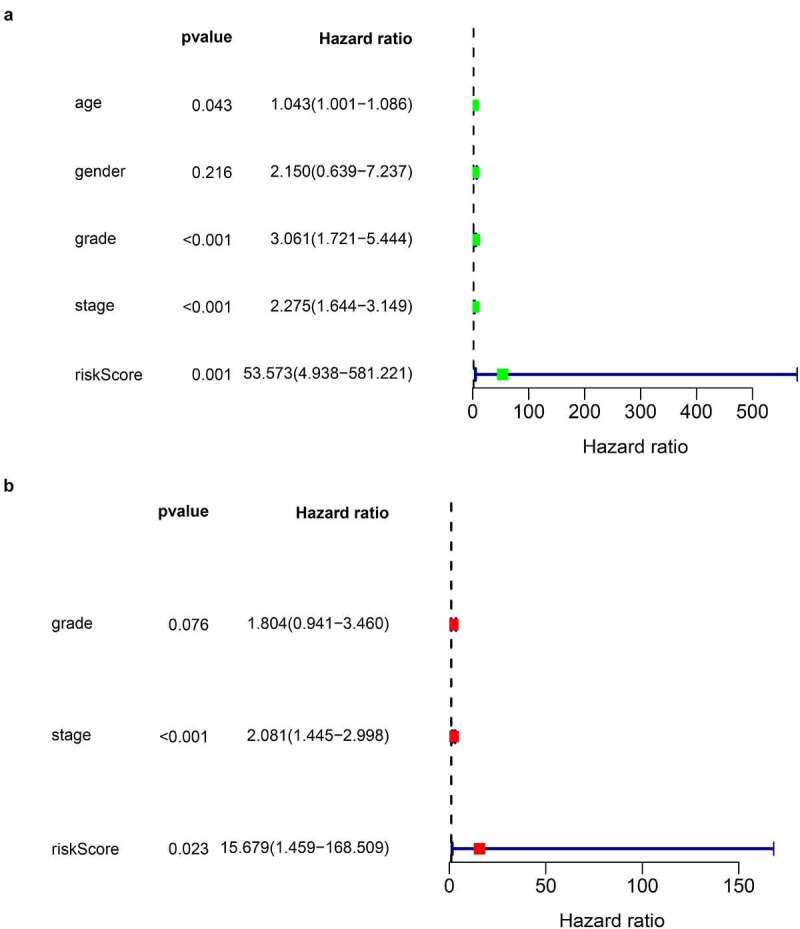
Univariate and multivariate Cox regression analysis forest plot of validation cohort 2

**Figure 8.**
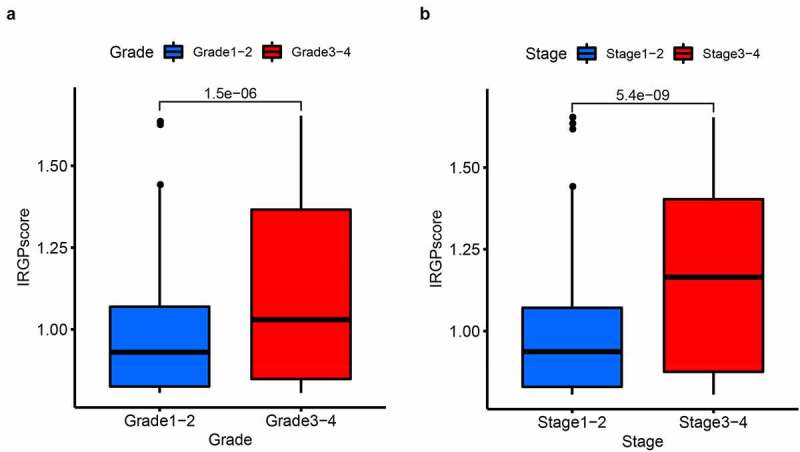
IRGP score in different clinicopathological factors

In all cohorts, the OS of the high-risk group was worse than that of the low-risk group (Figure 3). The p values of training cohort and validation cohort 1 were both less than 0.001, and the p-value of validation cohort was 0.005. Through ROC analysis of the three cohorts, we found that the 1-year, 3-year, and 5-year AUC values of the training cohort were 0.801, 0.809, and 0.834, respectively. Similarly, the AUC values of validation cohort 1 were 0.69, 0.672, and 0.728 for 1, 3, and 5 years, respectively. The AUC values of validation cohort 2 were 0.717, 0.768, and 0.778 for 1, 3, and 5 years, respectively (Figure 4). In a forest plot of training cohort, the p-values of grade, stage, age, gender, and risk score were all less than 0.05 in single factor cox analysis, and the risk score had the largest hazard ratio (HR) value. In order to be more accurate, we put the indicator with p-value less than 0.001 in the single factor cox analysis into multi-factor cox analysis and found that independent prognostic factors contain both risk score and stages and the HR value of each one was greater than 1 (Figure 5). The forest plot of two validation cohorts verified the reliability of our results (Figure 6 and Figure 7). It was found that the IRGP Score of the high-stage group and the high-grade group is much higher than their corresponding low groups (Figure 8).

(a) Training cohort. (b) Validation cohort 1. (c) Validation cohort 2.

(a) 1 year ROC curve of training cohort. (b) 3 year ROC curve of training cohort. (c) 5 year ROC curve of training cohort. (d) 1 year ROC curve of validation cohort 1. (e) 3 year ROC curve of validation cohort 1. (f) 5 year ROC curve of validation cohort 1. (g) 1 year ROC curve of validation cohort 2. (h) 3 year ROC curve of validation cohort 2. (i) 5 year ROC curve of validation cohort 2.

(a) univariate Cox regression analysis forest plot of training cohort. (b) multivariate Cox regression analysis forest plot of training cohort.

(a) univariate Cox regression analysis forest plot of validation cohort 1. (b) multivariate Cox regression analysis forest plot of validation cohort 1.

(a) univariate Cox regression analysis forest plot of validation cohort 2. (b) multivariate Cox regression analysis forest plot of validation cohort 2.

(a) Grade. (b) Stage.

### Construction and validation of nomogram prognostic model

**Figure 9.**
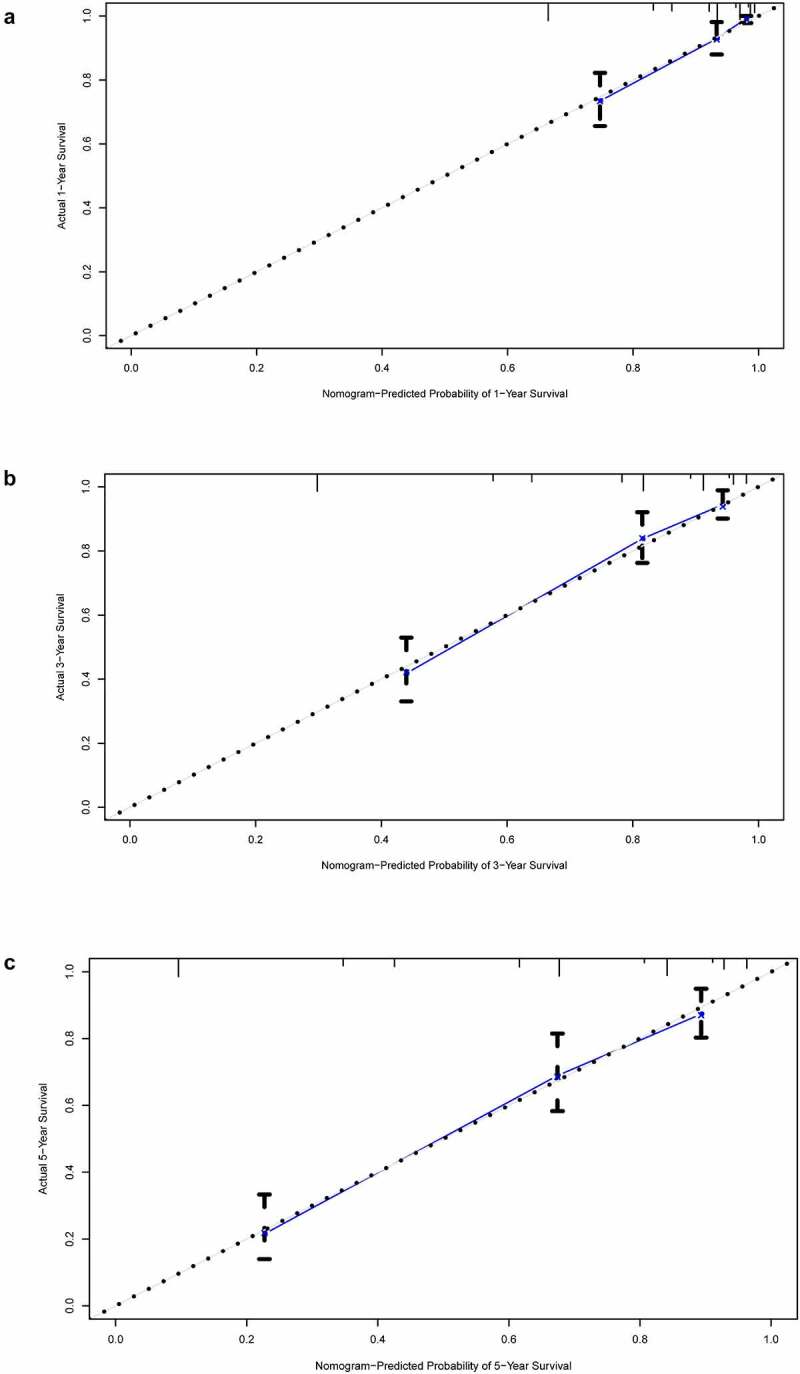
Nomogram model constructed by riskscore and stage predicting 1, 3 and 5 year OS for ccRCC patients

**Figure 10.**
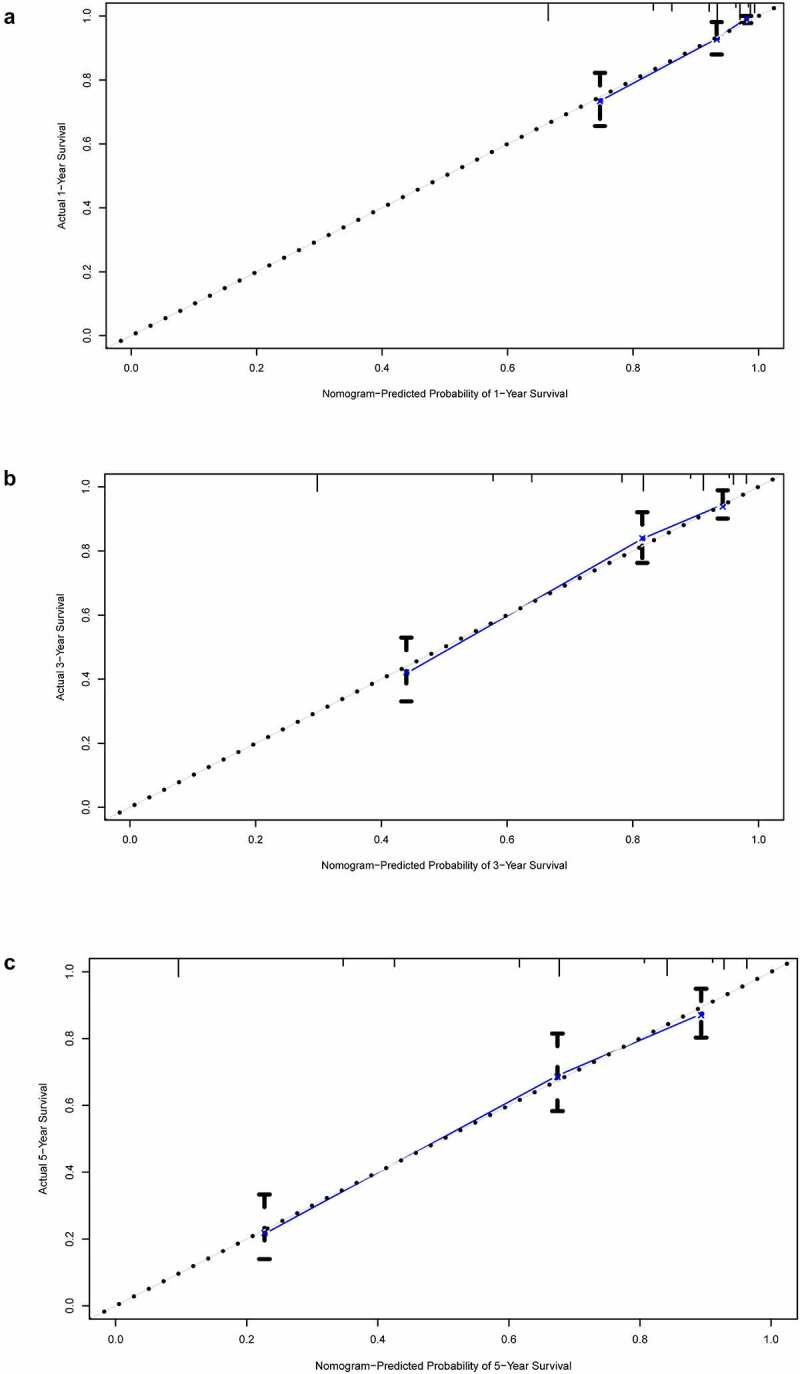
Calibration curve of nomogram model

**Figure 11.**
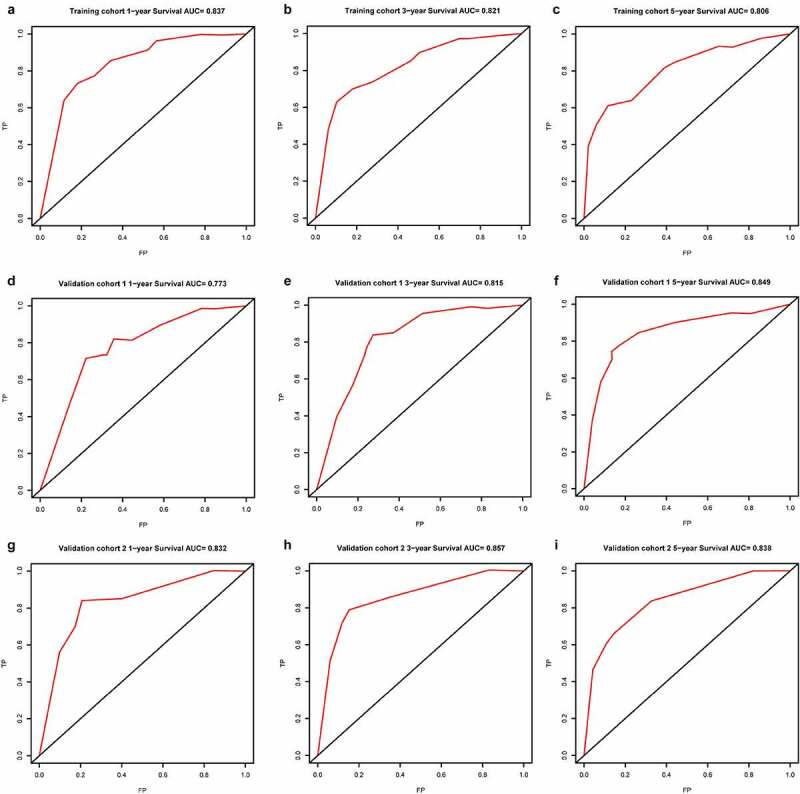
1, 3, and 5-year ROC curve of the three cohorts

**Figure 12.**
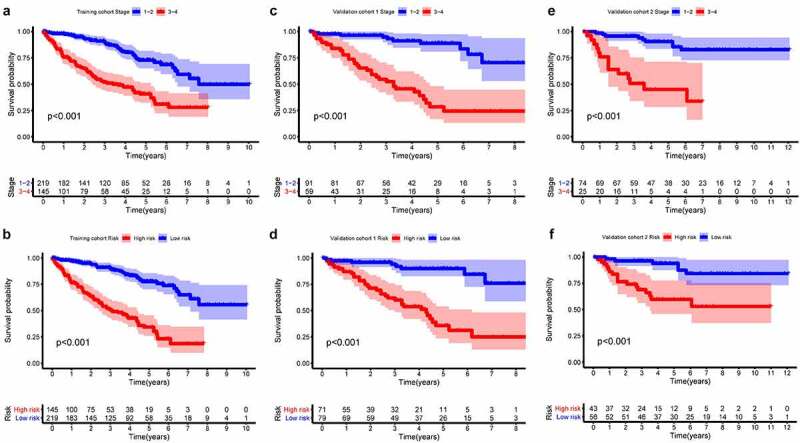
Survival plot of the three cohorts

In the former analysis, we found that both stage and risk score can be independent prognostic factors and that their HR value was greater than 1. Based on these factors, a nomogram was established to predict the 1-year, 3-year, and 5-year OS (Figure 9). The C-index was 0.7864. The OS calibration curves for 1, 3, and 5 years were in high accordance with observation results (Figure 10). Through ROC analysis of the three cohorts, we found that AUC values of the training cohort were 0.837, 0.821, and 0.806 for 1, 3, and 5 years, respectively. The AUC values of validation cohort 1 were 0.773, 0.815, and 0.849 for 1, 3, and 5 years, respectively. The AUC values of validation cohort 2 were 0.832, 0.857, and 0.838 for 1, 3, and 5 years, respectively (Figure 11). In all cohorts, the OS of the low-risk group was better (Figure 12). The p values of the three cohorts were both less than 0.001.

(a) 1-year calibration curve. (b) 3-year calibration curve. (c) 5-year calibration curve.

(a) 1 year ROC curve of training cohort. (b) 3 year ROC curve of training cohort. (c) 5 year ROC curve of training cohort. (d) 1 year ROC curve of validation cohort 1. (e) 3 year ROC curve of validation cohort 1. (f) 5 year ROC curve of validation cohort 1. (g) 1 year ROC curve of validation cohort 2. (h) 3 year ROC curve of validation cohort 2. (i) 5 year ROC curve of validation cohort 2.

(a) survival plot of stage in training cohort. (b) survival plot of united-risk model in training cohort. (c) survival plot of stage in validation cohort 1. (d) survival plot of united-risk model in validation cohort 1. (e) survival plot of stage in validation cohort 2. (f) survival plot of united-risk model in validation cohort 2.

### Analysis of immune cell infiltration in different groups

**Figure 13.**
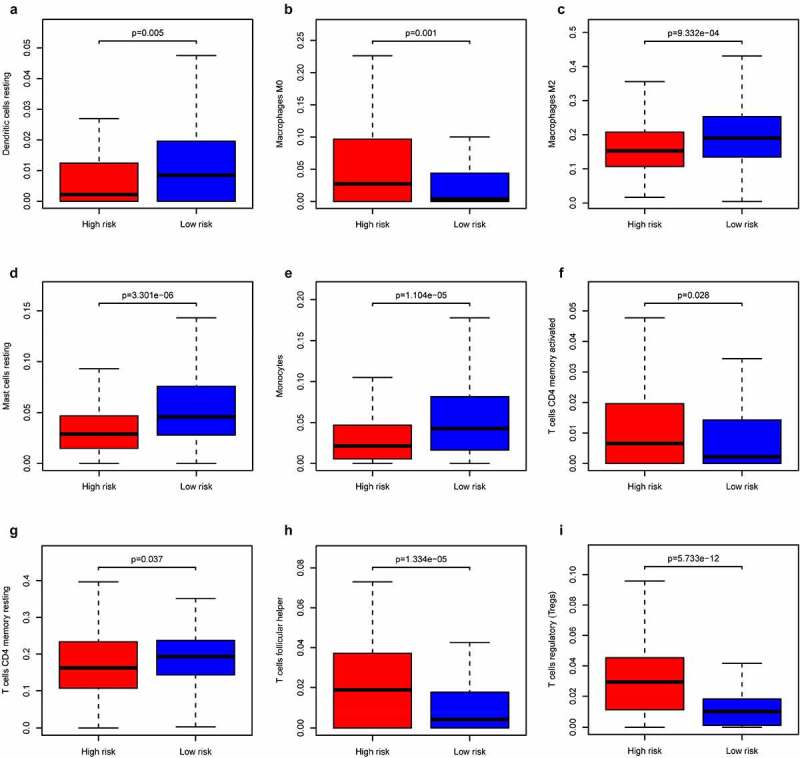
Immune infiltration status of training cohort

**Figure 14.**
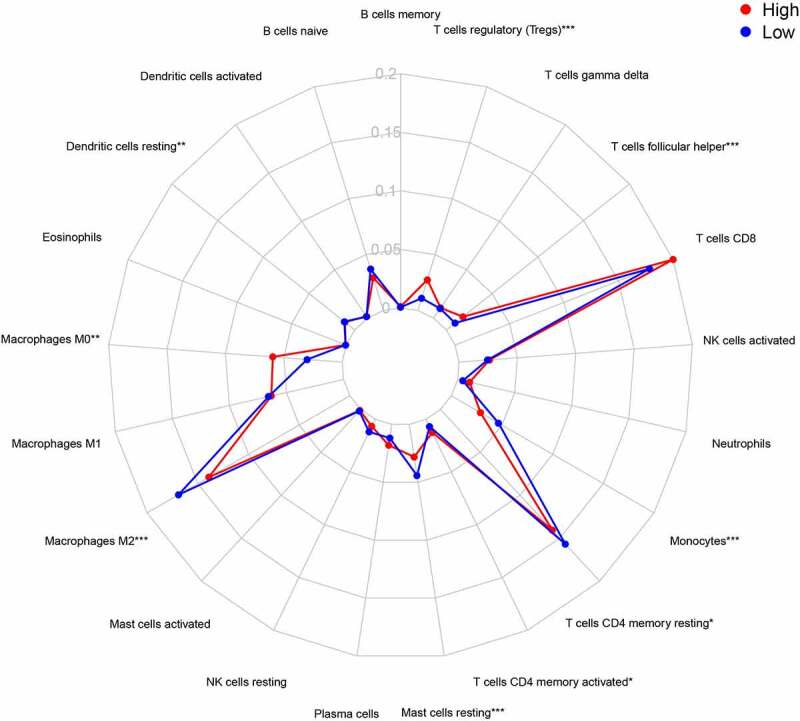
Summary of the 21 immune cells’ abundance for different risk groups of training cohort

Research has shown that immune cell infiltration is associated with the prognosis of tumors [27]. CIBERSORT was applied to analyze the infiltration of 21 immune cells in the two risk groups in the training cohort. As shown in Figure 10 and Figure 11, the number of macrophages M0, CD4 memory activated T cells, T follicular helper cells, and T regulatory cells (Tregs) was higher in the high-risk group. Moreover, the number of resting dendritic cells, macrophages M2, resting mast cells, monocytes, and resting CD4 memory T cells was higher in the low-risk group than in the high-risk group (Figure 13 and Figure 14).

(a) Dendritic cells resting. (b) Macrophages M0. (c) Macrophages M2. (d) Mast cells resting. (e) Monocytes. (f) T cells CD4 memory activated. (G) T cells CD4 memory resting. (h) T cells follicular helper. (i) T cells regulatory (Tregs).

## Discussion

Kidney cancer is the 14^th^ most common malignant tumor in the world, accounting for 3.5% of all human malignancies. It is the most dangerous disease among urological cancers. Limited by its characteristics, the most effective therapy for kidney cancer is still surgery [28]. However, not all patients can tolerate surgical treatment, and the effect of surgical treatment still has its limitations. Therefore, it is very important to develop other treatments, and immunotherapy may be a breakthrough point. Recently, it has been found that immunotherapy plays an excellent role in a variety of cancers [29–31].

At present, some researchers are exploring the association between IRGs and kidney cancer. Yong Zou et al. acquired expression data from TCGA database and used the LASSO-COX method to establish a model based on 14 IRGs after 1,000 iterations to assess the prognosis of patients [16]. However, the model lacks validation data from other platforms and it has platform bias. Feng et al. analyzed the expression of CASR, COL4A1, GPR4, MMP2, DCN, UTS2, and LDLR genes, which may be applied to improve RCC diagnosis and considered potential treatment targets, using information from Gene Expression Omnibus database with the help of Gene Set Enrichment Analysis, Gene Ontology, Kyoto Encyclopedia of Genes and Genomes, and Protein–protein Interaction method [32]. There is also a study based on the bioinformatic analysis of pRCC in which a prognostic model was constructed based on IRGPs, but this model does not include clinical factors [17]. The methods used for other cancers, such as cutaneous melanoma, are similar to our methods. However, they are based on too many gene pairs, which will be relatively cumbersome in clinical applications [33].

At present, due to the usage of different platforms and the differences in samples, gene expression data in public databases are biased, which poses a challenge for accurate analysis. The usual approach is to standardize the data. In this study, we avoided the impact of different platforms by calculating the ratio of expression values between different genes in the same sample, and the data did not need to be standardized. Recently, this method has also been used in some other studies and has good reliability [21,34].

In this study, a new united prognostic signature was developed for ccRCC based on 11 IRGPs combined with clinical stage, and it was verified on two platforms and three datasets, which strongly proved the effectiveness of the model. We think that construction of prognostic models should not be limited to gene expression, and clinicopathological factors should also be taken into account, which will make the models more comprehensive and reliable.

Immune cell infiltration analysis of samples revealed that the number of macrophages M0, CD4 memory activated T cells, Tregs, and T follicular helper cells was higher in the high-risk group. Furthermore, the number of macrophages M2, resting dendritic cells, resting mast cells, monocytes, and resting CD4 memory T cells was higher in the low-risk group. Blood lymphocytes have been proven to resist cancer cells in the host. Decreased lymphocyte counts are associated with poor prognosis for many types of cancer, including RCC [35–37]. However, T follicular helper cells and Tregs are considered as factors that promote cancer progression and they are associated with poor prognosis of patients [38,39]. This is consistent with our analysis results. Besides, a study has shown that in bladder cancer, CD4 memory activated T cells are related to the good prognosis of patients. Therefore, further research is needed to explore the relationship between them and ccRCC [9]. Monocytes that infiltrate tumor tissues also have an impact on the development and progression of cancers [40]. The value of macrophages/monocytes in the formation and progression of malignant cancers is still controversial because they inhibit or increase the potential of monocytes in malignant tumors [41]. The role of mast cells and dendritic cells in ccRCC and their relationship with tumor angiogenesis in renal cancer is not yet clear [42], and further research is needed. Overall, existing studies have confirmed our results and provided directions for future research.

However, our research has some limitations. First, our united risk model is based on the bioinformatics analysis of TCGA and ArrayExpress. To be more reliable, we need to combine clinical specimens and evaluate them through experimental methods such as qRT-PCR in the future. Secondly, although we did everything we could to eliminate bias and errors, the data used to build the model came from retrospective studies, while prospective studies are more convincing. Furthermore, the datasets we used were from different platforms, with slightly different conditions, and possibly different stability and efficiency of the samples. Therefore, in the follow-up research, we will combine clinical samples, data, and various experimental methods to perform more extensive verification through a large amount of data.

## Conclusions

Calculating the ratio of expression values between different genes in the same sample can reduce the bias caused by platform differences. In this study, a new united prognostic signature was developed for ccRCC based on 11 IRGPs combined with clinical stage, and it was verified on two platforms and three datasets, which strongly proved the effectiveness of the model. Our results could help predict the prognosis of patients; however, detailed mechanisms remain to be explored.

## Data Availability

The expression and clinical information data used to support the findings of this study have been deposited in the TCGA (https://portal.gdc.cancer.gov/) and Arrayexpress(https://www.ebi.ac.uk/arrayexpress/) repository. The immune-related genes list was achieved from the Immport Shared Gene Lists Data(https://www.immport.org/home). The data of all the above databases can be downloaded freely, and our research complied with the agreement of these databases.

## Research highlights

·Paired comparison of immune gene expression values effectively reduces platform bias.

·Immune-related gene pairs were combined with stage to better predict the prognosis.

·Immune cell infiltration plays a role in the occurrence and development of tumors.
